# Clinical Characteristics of Diagnosis for Internet Gaming Disorder: Comparison of DSM-5 IGD and ICD-11 GD Diagnosis

**DOI:** 10.3390/jcm8070945

**Published:** 2019-06-28

**Authors:** Yeong Seon Jo, Soo Young Bhang, Jung Seok Choi, Hae Kook Lee, Seung Yup Lee, Yong-Sil Kweon

**Affiliations:** 1 Department of Psychology, Sung-Shin women’s University, Seoul 02844, Korea; 2 Department of Psychiatry, The Catholic University of Korea Uijeongbu St. Mary’s Hospital, Gyeonggi 11765, Korea; 3 Department of Psychiatry, Eulji Hospital, Eulji University College of Medicine, Seoul 01830, Korea; 4 Department of Psychiatry, SMG-SNU Boramae Medical Center, Seoul 07061, Korea; 5 Department of Psychiatry and Behavioral Science, Seoul National University College of Medicine, Seoul 03080, Korea; 6 Department of Psychiatry, Uijeongbu St. Mary’s Hospital, The Catholic University of Korea College of Medicine, Gyeonggi 11765, Korea; 7 Department of Psychiatry, Eunpyeong St. Mary’s Hospital, The Catholic University of Korea College of Medicine, Seoul 03312, Korea

**Keywords:** internet gaming disorder, gaming disorder, DSM-5 diagnosis criteria, ICD-11 diagnosis criteria, children and adolescents, clinician interview

## Abstract

The American Psychiatric Association (APA) included internet game disorder (IGD) in section III of the Diagnostic and Statistical Manual of Mental Disorders-Fifth Edition (DSM-5) on the condition that it guaranteed more clinical research and experience. The World Health Organization (WHO) also included Game Disorder (GD) in the 11th final revision of the International Classification of Diseases (ICD-11) and recently recognized it as a diagnosis code. This study aims to compare clinical characteristics and gaming behavior patterns between the IGD diagnosis criteria proposed by the DSM-5 and the GD diagnosis criteria proposed by the ICD-11 based on clinical cohort data (c-CURE: clinic-Cohort for Understanding of internet addiction Rescue factors in Early life) obtained in the Republic of Korea. Psychologists and psychiatrists conducted semi-structured interviews with children/adolescents and their caregivers to identify IGD (Diagnostic Interview for Internet, Game, SNS, etc. Addiction, DIA), and comorbid psychiatric disorders (Kiddie-Schedule for Affective Disorders and Schizophrenia-Present and Lifetime Version-Korean version, K-SADS-PL). The cohort was divided into three IGD diagnosis groups (Normal, DSM5, DSM5 + ICD11) based on DSM-5 and ICD-11 diagnosis criteria. Internet usage pattern and addiction characteristics and psychiatric comorbidities were compared among the three IGD diagnosis groups. The Normal group consisted of 115 subjects, the DSM5 group contained 61 subjects, and the DSM5 + ICD11 group amounted to 12 subjects. The DSM5 + ICD11 group had a lower age of starting use of Internet/games/smartphones than other groups and the average time of Internet/game/smartphone use during weekdays/weekends was the highest. Also, in the eight items scored, excluding ‘deceiving’ and ‘craving’, the rate of threshold was highest in the DSM5 + ICD11 group, followed by the DSM5 group and the Normal group. On the other hand, ‘deceiving’ and ‘craving’ were the highest in DSM5, followed by DSM5 + ICD11 and Normal. The DSM5 + ICD11 group had significantly higher rates of depressive disorder, oppositional defiant disorder (ODD) and conduct disorder (CD) compared to other groups. This study provides implications for the clinical characteristics of IGD diagnosis in the field by comparing the DSM-5 IGD diagnosis criteria with the ICD-11 GD diagnosis criteria. Furthermore, this study provides empirical evidence that ICD-11 GD emphasizes serious symptoms such as functional impairment caused by excessive Internet/game/smartphone use over a long time, and it supports the validity of the ICD-11 GD diagnosis.

## 1. Introduction

In today’s modern society, which can be characterized as an information society, Internet use is inevitable. The universalization of the Internet has positive aspects such as convenience, informatization, and diversification. On the other hand, excessive use of Internet can have a dysfunctional effect on various areas and lead to Internet addiction. In the Republic of Korea, the prevalence of Internet/smartphone addiction risk has continuously increased from 13.1% to 21.8% in 2013–2018 (high risk: 1.3%→2.7%, mild risk: 11.8%→19.1%) [1]. Recently, not only Internet/game use but also smartphone use has become a social issue. By age group, the proportion of Internet/smartphone addiction risk was 29.3% (high risk: 3.6%, mild risk: 25.7%) for adolescents, 20.7% (high risk: 2.0%, mild risk: 20.7%) for children, and 18.1% (high risk: 2.7%, mild risk: 18.1%) for adults [1]. In other words, children and adolescents have the greatest risk of Internet/smartphone addiction. In particular, the risk of children’s Internet/smartphone addiction increased steeply from 12.4% to 20.7% in 2015–2018 [1]. As such, the age of Internet/smartphone addiction risk groups is steadily decreasing, and excessive use of Internet/smartphones by children and adolescents is considered to be a serious social problem.

In 2013, the American Psychiatric Association (APA) determined that Internet gaming disorder (IGD) requires further research and data accumulation, and so included IGD in section III of the Diagnostic and Statistical Manual of Mental Disorders-Fifth Edition (DSM-5) [2]. Since 2014, the World Health Organization (WHO) has been responding to game addiction as an important public health problem [3]. Game addiction was defined as gaming disorder (GD) and characterized by specific diagnosis criteria [4]. The 11th final revision of the International Classification of Diseases (ICD-11) which included GD was released in June 2018 [5]. The DSM-5 and ICD-11 both defined GD as being characterized by a pattern of repetitive or persistent gaming behavior [2,5]. The game behavior and the characteristics that meet the diagnosis criteria are normally evident over a period of at least 12 months [2,5].

In the DSM-5, five of the nine diagnosis criteria (preoccupation or obsession, withdrawal, tolerance, loss of control, loss of interest, continued overuse, deceiving, escape of negative feelings, functional impairment) must be met within a year to be diagnosed as IGD [2]. On the other hand, in the ICD-11, to be officially diagnosed with GD, a patient must exhibit three symptoms (impaired control over gaming, increasing priority given to gaming, continuation or escalation of gaming despite the occurrence of negative consequences) [5]. In the ICD-11, behavioral addiction such as gambling disorder and GD is classified as an addictive disorder, so the name is changed from ‘dependence’ to ‘use disorder’ [5]. Therefore, biological concepts such as withdrawal and tolerance proposed in the IGD of the DSM-5 is excluded from the ICD-11 GD diagnosis criteria. Withdrawal and tolerance are a physiological response that occurs during the process of adapting neurons when addictive substances act on brain nerves [6]. The concept of diagnosis is comprehensively defined as a functional impairment that shows pathological aspects rather than biological definition [6].

During the 72nd World Health Assembly in Geneva, Switzerland, the entire WHO board voted unanimously to induct the disease into their officially listed health risks. The WHO officially voted to recognize GD as a medical disease on May 25, 2019 [7]. However, since clinical studies on GD are lacking and diagnostic consensus is ambiguous, it was still argued that GD is not based on sufficient scientific evidence to justify its inclusion in one of the WHO’s most important norm-setting tools [8,9,10,11,12]. Also, there are concerns that social prejudice and negative perceptions of games may lead to a stigma of ‘mental disorder’ [8,9,10,11,12].

Alcohol use disorder is usage of alcohol, which means that the pattern is ‘morbid’ and ‘addictive’. Likewise, GD means that the game usage pattern is ‘morbid’ and ‘addictive’. Not all drinkers are addicted, and games do not reach everyone’s addiction. That is, classifying GD as a disorder means that game is used as ‘maladaptive or pathological’ rather than making value judgments such as ‘good or bad’ in the game itself [6]. Most Internet/game/smartphone users use the Internet/game/smartphone as a leisure and entertainment activity. However, for excessive and pathological users who are classified as having gaming disorders, medical-health services including socially accurate diagnosis and treatment are needed [8,9,10,11,12].

In 2012, studies were published comparing the volume of brain parts by dividing healthy comparison subjects, professional game players, and game addiction patients into three groups [13]. The study found increased gray matter volumes of the left cingulate gyrus in professional game players and increased gray matter volumes in the left thalamus in game addiction patients. This means that professional game players conduct intensive training under systematic and controlled plans, while game addiction patients do not control brain impulses and lack the ability to manage the frontal lobe. Game addiction patients and professional gamers play games for a long time, but the results are different. That is, it is proven that the structure of the brain has changed. Also, in 2018, a follow-up study of 755 IGD patients who received IGD clinical treatment over five years was published [14]. The recovery rate of 367 patients who were treated with IGD for eight weeks and who completed the follow-up study was confirmed. The results showed that, two-thirds of patients had not fully recovered, and experienced ongoing difficulties. However, those who started the game late or started treatment recovered relatively quickly. In particular, since children and adolescents are at a stage of development, excessive use of the Internet/games/smartphones is likely to hinder brain development as well as functional impairment if it lasts for a long time [13,15].

Classifying GD as a disorder does not deny the game itself, but rather is seen as a step toward early intervention in excessive game use. Furthermore, by providing a guideline to enjoy the Internet/games/smartphones properly as leisure and entertainment activities, patients will be able to establish such activities as part of a healthier life hobby. ‘Denial’ is one of the major characteristics of addiction [16], and children and adolescents tend to underestimate negative aspects due to the influence of ‘social desirability’. In fact, in a study comparing self-reports and clinician interview diagnosis for IGD showed that the false-positive rate was 9.6% and the false-negative rate was 44% in adolescents [17]. A comparison of IGD diagnosis between the DSM-5 assessments and psychiatric interviews revealed diagnostic differences [18]. As the severity of IGD symptoms increased, it was found to be associated with functional impairment [19].

Therefore, this study aims to compare clinical characteristics and gaming behavior patterns between the IGD diagnosis criteria proposed by the DSM-5 and the GD diagnosis criteria proposed by the ICD-11 based on the clinical data obtained by psychologists and psychiatrists in face-to-face interviews with children/adolescents and their primary caregivers, not through self-reports. It was confirmed that the ICD-11 GD is more related to serious Internet/game/smartphone addiction than IGD diagnosis proposed by the DSM-5 and applies strict diagnosis criteria. This study provides basic data on clinical characteristics of IGD diagnosis and suggests the validity of future diagnosis of the ICD-11 GD.

## 2. Materials and Methods

### 2.1. Participants and Procedure

**Figure 1:**
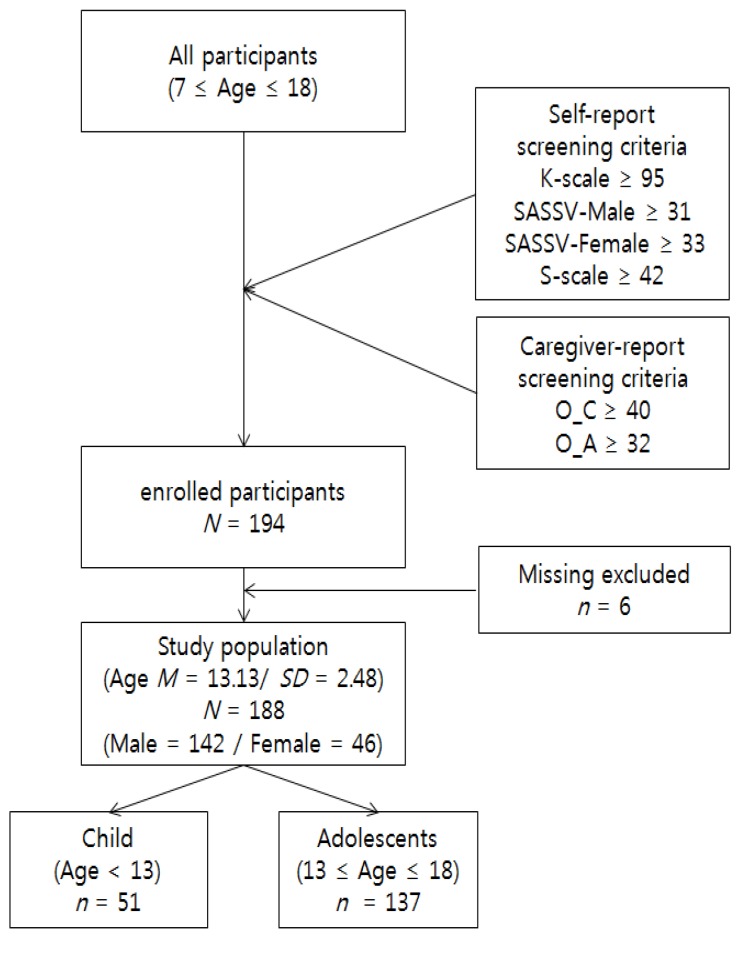
Study flow chart and socio-demographic characteristics. Note: the cut-off values for each screening scale is presented. K-scale = Korean Scale for Internet Addiction for adolescents; SAS-SV = Smartphone Addiction Scale-short form version; S-scale = Korean Smartphone Addiction scale; O_C = Internet Addiction Proneness Scale for Child checked by caregivers; O_A = Internet Addiction Proneness Scale for Adolescents checked by caregivers.

From 2015 through 2019, a multicenter clinical cohort study (c-CURE: clinic-Cohort for Understanding of Internet addiction Rescue factors in Early life) was conducted in the Seoul metropolitan area in the Republic of Korea. The c-CURE was designed to explore risks and protective factors for Internet/game/smartphone addiction and their natural courses among children and adolescents of the clinic cohort. Figure 1 shows a flow chart for the c-CURE study.

All participants (children or adolescents, and their primary caregivers) conducted a screening questionnaire on Internet and smartphone addiction (K-scale: Korean Scale for Internet Addiction for adolescents [20]; SAS-SV: Smartphone Addiction Scale-short form version [21]; S-scale: Korean Smartphone Addiction scale [22]; O_C: Internet Addiction Proneness Scale for Child checked by caregivers [23]; O_A: Internet Addiction Proneness Scale for Adolescents checked by caregivers [24]). Subjects who received higher scores than the cutoff on at least one scale measuring the Internet/smartphone addiction questionnaires were enrolled in the c-CURE. A total of 194 children and adolescents were enrolled, but six participants who did not participate in the diagnostic interview for Internet/game/social network service (SNS) addiction were excluded from the study. Therefore, data from a total of 188 subjects were analyzed. Of the 188 participants (mean age, 13.13 ± 2.48 years), 142 were males and 46 were females.

Clinical psychologists and psychiatrists conducted semi-structured face-to-face interviews with each participant and their primary caregivers to identify Internet/game/smartphone addiction (DIA) and comorbid psychiatric disorders (K-SADS-PL). For the validity and reliability of the diagnosis evaluation, all the mental health professionals involved in the clinical interviews underwent the same training with regard to the protocol.

### 2.2. Measures

#### 2.2.1. Diagnostic Interview for Internet, Game, SNS, etc. Addiction (DIA)

The diagnostic interview for Internet, game, SNS, etc. addiction (DIA) is a semi-structured interview diagnostic tool developed by adding ‘craving‘ to existing domains in the DSM-5 section III Internet game disorder (IGD)-9 criteria [25]. Each item was scored on a 0–3 Likert scale (0: no information; 1: no symptoms; 2: below threshold; 3: above threshold), and the number of items with a score of 3 was calculated as the total DIA score (range: 0–10) [25].

Interviews about DIA and Internet/game/SNS usage took about 20–30 min for each child/adolescent and their primary caregivers. Each DIA item was designed with standardized representative questions and various detailed examples (Table 1). For example, the following representative questions and examples are presented to assess the ‘cognitive salience’ which indicates preoccupation/obsession with Internet/game/SNS. The representative question is “Even if you are not using the Internet/game/SNS, do you spend a lot of time thinking about Internet/game/SNS or planning what to do next?”, and various detailed examples are “ I often think and plan about activities such as the Internet/game/SNS in the past or the future.”; “Internet/game/SNS is a main activity during daily life”; “The content (such as scenes/music) associated with the usual Internet/game/SNS is often brought up and engrossing at that time”; “I am often immersed to the point that I forget the time while using the Internet/game/SNS”; and so on. Clinical psychologists and psychiatrists determined whether the subject was addicted to the Internet/game/SNS by combining the answers of each participant and their primary caregiver interviews with the clinician’s judgment. In addition, we asked questions related to Internet usage such as Internet contents, Internet devices used, and time spent on the Internet/games/smartphones, and so on.

#### 2.2.2. Diagnostic Interview for Comorbid Psychiatric Disorders

The Kiddie-Schedule for Affective Disorders and Schizophrenia-Present and Lifetime Version-Korean version (K-SADS-PL) is a psychiatric disorders diagnostic interview tool. The validity and reliability of this tool was verified in 2003 [26]. K-SADS-PL involves semi-structured interviews, taking about 20–30 min for each child/adolescent and their primary caregivers. K-SADS-PL was developed based on the DSM-III-R and DSM-IV psychiatric disorders criteria [26]. Each diagnosis criterion of the psychiatric disorders was scored on 0–3 Likert scale (0: no information; 1: no symptoms; 2: below threshold; 3: above threshold). The number of items with a score of 3 was calculated as the total score according to the diagnosis criteria for each psychiatric disorder, and the existence or nonexistence of each psychiatric disorders was thus decided [26]. Clinical psychologists and psychiatrists determined whether the subject had psychiatric disorders by combining the answers of each participant and their primary caregiver interviews with the clinician’s judgment.

### 2.3. Data Management and Analysis

The IGD diagnosis cohort was divided into three groups based on the number of 3 point scores, indicating the threshold of the DIA item. The DSM-5 IGD diagnosis includes more than five among the DIA 1–9 items and the ICD-11 GD diagnosis includes all DIA 4, 5, 6, and 9 items.

The subjects that matched with the IGD diagnosis criteria proposed by the DSM-5 formed the ‘DSM5′ diagnosis group [2]. The participants that met the GD diagnosis criteria proposed by the ICD-11 formed the ‘ICD11′ diagnosis group [5]. However, since the ICD11 diagnosis group also met the DSM-5 IGD diagnosis criteria, they were named the ‘DSM5 + ICD11’ group. If participants did not meet IGD criteria at all, they included in the ‘Normal’ group.

Chi-squared tests and variance analysis (ANOVA) were conducted using SPSS software version 23.0 (SPSS, Inc., Chicago, IL, USA). For the nominal variables, the chi-squared test was used to analyze frequency differences among the three groups. While for the continuous variables, variance analysis was used to verify mean differences among the three groups. If statistically significant differences were identified in the variance analysis, then post hoc analysis was also performed. Through this analysis, we tried to compare the Internet usage pattern and addiction characteristics, as well as psychiatric comorbidities among the three IGD diagnosis groups. The following research problem and hypothesis was formed: There will be differences in Internet usage, addiction characteristics, and psychiatric comorbidities among the three groups (Normal, DSM5, DSM5 + ICD11). In particular, the DSM5 + ICD11 group will have more severe Internet addiction symptoms than the DSM5 and Normal group.

### 2.4. Ethical Approval

The study was conducted in accordance with the Declaration of Helsinki. The study was approved by the Institutional Review Board (IRB) for human subjects of the Catholic University of Korea Uijeonbu St. Mary’s Hospital (UC150NMI0072), Eulji University Seoul Eulji Hospital (EMCS2015-05-020-001), and Seoul Metropolitan Government Seoul National University Boramae Medical Center (16-2016-4). Written consent was received from all children/adolescents and their primary caregivers. The participants were provided copies of this consent, including detailed explanation of participation in the study such as confidentiality and freedom of choice to participate.

## 3. Results

### 3.1. Comparison of DSM-5 IGD Diagnosis and ICD-11 GD Diagnosis

Table 2 presents the number of cases according to the DSM-5 IGD diagnosis and the ICD-11 GD diagnosis. A total of 73 children and adolescents (38.8%) were diagnosed according to the IGD diagnosis criteria proposed by the DSM-5, of whom 58 were males and 15 were females. A total of 12 children and adolescents (6.4%) were diagnosed according to the GD diagnosis criteria proposed by the ICD-11, and all were male. They also met the IGD diagnosis criteria proposed by the DSM-5. In addition, we examined the number of DIA 3 point scores corresponding to IGD diagnosis criteria proposed by the DSM-5 in 12 subjects who met the GD diagnosis criteria of the ICD-11. It was found that 3 subjects had 6 symptoms, 2 subjects had 7 symptoms, 4 subjects had 8 symptoms, and 1 subject had 9 symptoms. Overall, the number of IGD symptoms suggested by the DSM-5 was high. A total of 115 children and adolescents (61.2%) were in the Normal group, as they did not meet the IGD diagnosis criteria proposed by the DSM-5 and ICD-11. Among these, 84 were male and 31 were female. Therefore, the DSM5 + ICD11 group had 12 subjects, the DSM5 group had 61 subjects, and the Normal group had 115 subjects. There was no gender difference according to three diagnosis groups (*x*^2^(2) = 4.273, *p* = 0.118).

### 3.2. Clinical Characteristics of IGD Diagnosis Groups

Among the three diagnosis groups, the frequency of Internet use characteristics and psychiatric disorders are shown in Table 3 and Table 4. It was found that using two or more types of Internet content rather than using only one type of Internet content in three groups. Most of the subjects used games (primary: 82, secondary: 20) and video clips such as YouTube and Africa TV (primary: 24, secondary: 83). In particular, everyone in the DSM5 + ICD11 group used two or more types of Internet platforms (game (primary: 8, secondary: 2), video (primary: 2, secondary: 7)). Eighty-seven subjects (46.3%) used two or more types of Internet devices such as a smartphone and PC or tablet, and 77 subjects (41.0%) used a smartphone. It was found that most children and adolescents use Internet-enabled devices that are easy to carry.

There was a significant difference in the presence or absence of depressive disorder (*x*^2^(2) = 6.229, *p* < 0.05), oppositional defiant disorder, and conduct disorder (*x*^2^(2) = 31.160, *p* < 0.001). Four subjects in the DSM5 + ICD11 group (33.3%) had depressive disorder. On the other hand, 12 subjects in the DSM5 group (19.7%), 12 subjects in the Normal group (10.4%) had depressive disorder, and four subjects in the DSM5 + ICD11 group (33.3%) had ODD or CD. On the other hand, one subject in the DSM5 group (1.6%), and two subjects in the Normal group (1.7%) had ODD or CD. In other words, the DSM5 + ICD11 group had higher rates of depressive disorder, ODD, and CD compared with the other groups. Attention deficit hyperactivity disorder (ADHD) was the most common in all groups compared with other psychiatric disorders.

Internet using behaviors according to the three diagnosis groups are presented in Table 5. The DSM5 + ICD11 group had a lower age at which Internet/game/smartphone use started as compared to the other groups. However, there was no statistically significant difference among the three groups regarding the age of starting Internet use (*F*(2, 184) = 0.056, *p* = 0.945)/game use (*F*(2, 184) = 0.256, *p* = 0.774)/smartphone use (*F*(2, 184) = 1.734, *p* = 0.179). The average time of Internet/game/smartphone use during weekdays was the highest at 290.00 min/day in the DSM5 + ICD11 group, followed by 254.92 min/day in the DSM5 group, and the lowest at 248.78 min/day in the Normal group. However, there was no statistically significant difference among the three groups in terms of the average time of Internet use during weekdays (*F*(2, 185) = 0.412, *p* = 0.663). Also there was no statistically significant difference among the three groups in terms of the average time of Internet use on weekends (*F*(2, 185) = 2.626, *p* = 0.075). Still, it was confirmed that the DSM5 + ICD11 group spent the most time on the Internet/game/smartphone (over 477.50 min/day on weekends), compared to 400.82 min in the DSM5 group and 361.57 min in the Normal group. In the DSM-5 diagnosis group, weekday and weekend Internet/game/smartphone usage time differed from the Normal group by 6.14 min and 39.25 min.

**Figure 2:**
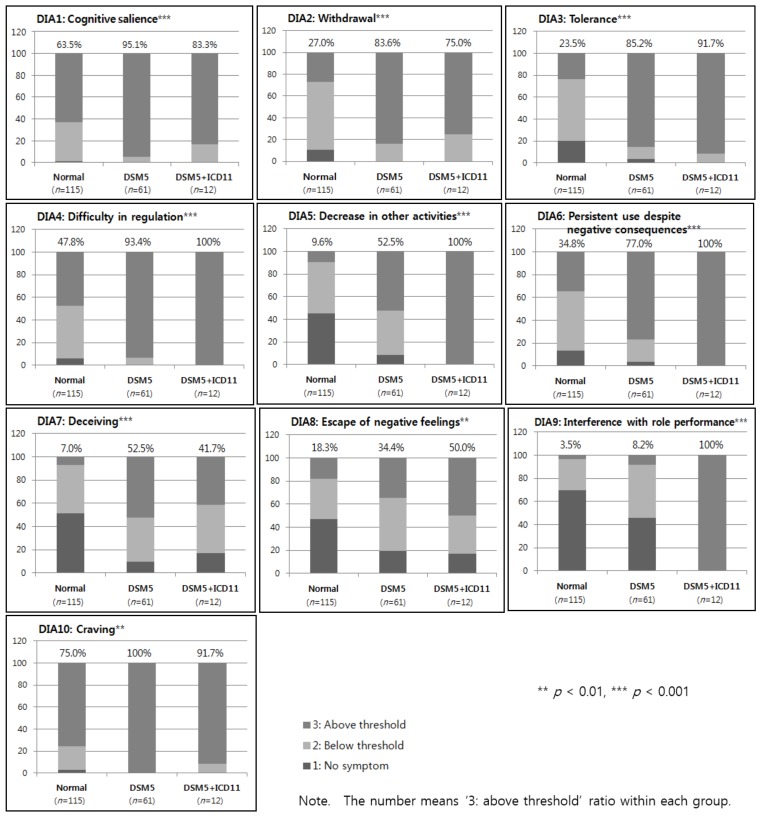
Score percentages of each DIA item within the three groups.

As a result of examining the differences among all of the DIA items according to the Normal, DSM5, and DSM5 + IGD11 groups, it was found that there were statistically significant differences among the three groups in all 10 DIA items. The endorsed response percentages for each item within each group is shown in Figure 2. In the eight items, excluding ‘deceiving’ (DIA7) and ‘craving’ (DIA10), the rate of DIA 3 point scores corresponding to the threshold was highest in the DSM5 + ICD11 group, followed by the DSM5 group and the Normal group. On the other hand, ‘cognitive salience’ (DIA1) and ‘deceiving’ (DIA7) were the highest in the DSM5 group, followed by the DSM5 + ICD11 group and the Normal group. In both the DSM5 and DSM5 + ICD11 groups, ‘difficulty in regulation’ (DIA4) and ‘craving’ (DIA10) was higher than 90%. In the Normal group, participants were not diagnosed with IGD, but ‘cognitive salience’ (DIA1) was 63.5% and ‘craving’ (DIA10) was 75.0%.

## 4. Discussion

This study compared clinical characteristics and gaming behavior patterns between the IGD diagnosis criteria proposed by the DSM-5 and the GD diagnosis criteria proposed by the ICD-11 based on clinical data obtained in the Republic of Korea. The main findings of this study and implications as well as limitations are as follows.

First, we diagnosed not only games but also various Internet contents such as video clips (e.g., YouTube and Africa TV), and SNS in this study. It was determined that 64% percent (120 out of 188) used more than two kinds of Internet platforms. As a result of asking about the first and second rankings of Internet platforms in the case of using two kinds of Internet platforms, the majority of children and adolescents responded with games (primary: 82, secondary: 20) and video clips (primary: 24, secondary: 83). In particular, everyone in the DSM5 + ICD11 group used two or more types of Internet content [game (primary: 8, secondary: 2) and video clips (primary: 2, secondary: 7)]. Also, in the interview process, children and adolescents responded that they watched video clips showing games on YouTube, Africa TV, and so on, as well as playing games directly. This seems to be a change due to the spread of smartphones and tablet PCs that are convenient to carry and are not restricted by time and place. As evidence of this, 22 children and adolescents (11.7%) used a PC only, but 77 children and adolescents (41.0%) used a smartphone, and 87 children and adolescents (46.3%) used two Internet devices such as a smartphone and a PC or tablet PC. That is, the majority of them used portable devices such as smartphones and tablet PCs. Furthermore, the DSM5 + ICD11 group had the lowest age of starting Internet/game/smartphone use. Internet devices such as PCs and smartphones constantly provide fast and immediate stimuli. In addition, DIA4 (difficulty in regulation: 93.4% vs. 100%) and DIA10 (craving: 100% vs. 91.7%) were higher than 90% in both the DSM5 and DSM5 + ICD11 groups. Both the DSM-5 IGD diagnosis criteria and the ICD-11 GD diagnosis criteria indicated that loss of control over game usage is basically considered an essential symptom. As amount and frequency of behaviors to play the game increase, the brain’s reward pathway (pleasure center) is activated, while the self-regulation gradually loses and leads to addiction [6,13,15]. If excessive use of the Internet/game/smartphone continues, the brain reacts only to immediate phenomena, while calm and subtle factors become unregistered. A magnetic resonance imaging (MRI) study was conducted to monitor the brains of 18 college students who spent about 10 h a day online in China [15]. These students had less gray matter in the brain’s thinking part than the control groups, who spent less than 2 h a day online [15]. It has been proven that using the Internet/games/smartphones over a long period physically changes the brain structure. Therefore, it is necessary to be aware of the need for proper use of the Internet/game/smartphone and develop a habit of using it appropriately.

Despite the fact that children and adolescents with a potentially higher risk of IGD were enrolled through self-report screening, only 12 out of the 188 children and adolescents (6.4%) met the GD diagnosis criteria proposed by the ICD-11. They had six to nine IGD symptoms suggested by the DSM-5 IGD diagnosis criteria. The DSM5 + ICD11 group showed a significantly higher rate of DIA9 (interference with role performance: 100% vs. 8.2%), DIA5 (decrease in other activities: 100% vs. 52.5%), and DIA6 (persistent use despite negative consequences: 100% vs. 77.0%) compared to the DSM5 diagnosis group. This suggests that the GD diagnosis criteria proposed by the ICD-11 emphasizes functional impairment compared to the IGD diagnosis criteria proposed by the DSM-5. Also, it has been confirmed that these symptoms tend to increase as IGD severity increases [19], which may be a key diagnosis criterion for detecting IGD.

In addition, it was found that the DSM5 + ICD11 group had a higher prevalence of ODD and CD compared to the other groups. ODD and CD are types of ‘disruptive, impulse-control, and conduct disorders’, which are related to self-regulation problems for emotion and behavior [2]. It seems to be understandable in that IGD is related to impulsivity and self-regulation [25,27,28,29]. Due to indiscriminate use of Internet/games/smartphones, subjects not able to perform given tasks properly have trouble with parents and teachers, and experience maladjustment in daily life. Therefore, IGD children and adolescents who meet the ICD diagnosis criteria are likely to experience functional impairment and have serious difficulties in daily life. ODD + CD diagnosis may be related to a more maladaptive functional state because of symptom characteristics such as rule violation, interpersonal conflict, and conflict with authority [2].

In the present study, there was a significant difference in the rate of depressive disorder among the different diagnosis groups. This is consistent with previous studies reporting that the association between depression and Internet addiction is high [25,30,31]. Depressive disorder is characterized by sad, empty, and negative changes that affect individual performance [2]. So, contrary to ODD and CD, depressive disorders can be related to functional impairments such as lack of or withdrawal from real-life activity and interpersonal relationships due to excessive Internet/game/smartphone use [30,31].

On the other hand, there was no statistically significant difference in the rate of ADHD among the diagnosis groups, though it was the highest in terms of total frequency. While ADHD is a characteristic risk group of the Internet/game/smartphone addiction, it is estimated that its impact on serious Internet/game/smartphone addiction symptoms is small [19,29,32]. Based on these results, it is possible to assume that if the addiction problem becomes serious, the surrounding environment and maladaptive tendencies will have an effect on the subject and contribute to the continuance of the behavior. Therefore, it is suggested that appropriate treatment and intervention are needed for children and adolescents with high risk IGD. As demonstrated in previous studies, early treatment or intervention results in a relatively rapid recovery [14].

Third, DIA1 (cognitive salience: 95.1%), DIA7 (deceiving: 52.5%), and DIA10 (craving: 100%) showed the highest rate in the DSM5 group. The IGD diagnosis criteria proposed by the DSM-5 appear to be interested in not only the apparent academic, social, and relational function impairment but also invisible internal psychological damage. In the DSM-5 group, weekday and weekend Internet/game/smartphone usage time differed from the Normal group by 6.14 min and 39.25 min, respectively, which is consistent with the ‘deceiving’ results. It can be interpreted that this is related to the ‘denial’ defense system, which is one of the characteristics of addiction [16]. In particular, the usage time of the Internet/games/smartphones on weekdays was only 6.14 min more than the Normal group. Children and adolescents may not have enough time to use the Internet/games/smartphones because they attend schools and academies on weekdays. However, since the time spent with the caregiver is less than on weekends, the caregiver does not know all of the daily life of children and adolescents, as confirmed in the interviews. Thus, it is suggested that children and adolescents may have responded by shortening their hours of use on weekdays compared to their actual use. In a study confirming the discordance between the self-report and clinical diagnosis of IGD, the discordance rate was 53.6% (false-positive: 9.6%, false-negative: 44%) [17]. Of these, adolescents giving false-negative answers showed similar results to IGD adolescents in the evaluation of psychological characteristics [17]. However, the false-negative adolescents under-reported game usage time and symptoms more than IGD adolescents [17].

Finally, the Normal group was not diagnosed with IGD, but the rate of reaching the threshold was significantly high in DIA1 (preoccupation of obsession: 63.5%) and DIA10 (craving: 75.0%). This is perhaps because only subjects who received higher scores than the cutoff on at least one scale for the Internet/smartphone addiction questionnaire were enrolled.

In other words, IGD diagnosis criteria were not met in the Normal group, but some diagnosis criteria were met, indicating that these children and adolescents may be included in the potential risk group. The results of this study have limited generalizability to non-clinical groups, as it does not include a control group because the subjects were registered through self-report screening. Moreover, this study is aimed at Korean children and adolescents, which should be considered when applying generalization to other countries or regions.

Nevertheless, this study is different from previous studies in that clinical psychologists and psychiatrists directly based their diagnoses on interviews with children, adolescents, and primary caregivers. Based on these data, we compared the clinical characteristics and game behavior patterns between the IGD diagnostic criteria proposed by DSM-5 and the GD diagnostic criteria proposed by ICD-11. Accordingly, this study has implications for the characteristics of IGD diagnosis in the clinical field. It was confirmed that ICD-11 GD is related to more serious Internet/game/smartphone addiction and has strict diagnosis criteria. That is, this study provides empirical evidence that the ICD-11 GD diagnosis emphasizes serious symptoms such as functional impairment caused by excessive Internet/game/smartphone use over a long time, and it supports the validity of ICD-11 GD diagnosis for future use.

However, the four DIA items (DIA 4, 5, 6, 9) are conceptually similar to the ICD-11 GD diagnostic criteria, but the criteria for symptom severity such as DIA 6 and 9 are unclear. Therefore, further studies on the severity criteria of functional impairment are needed.

In addition, since the psychosocial characteristics were not verified according to the IGD diagnosis groups, there is a limitation in not identifying the protection factors and risk factors of each diagnosis group. Thus, further studies are expected to provide data on the prevention and early intervention of IGD by comparing the psychosocial characteristics according to the IGD diagnosis groups.

## Figures and Tables

**Table 1:** Item content and standardized representative questions in Diagnostic Interview for Internet, Game, SNS, etc. Addiction (DIA).

Item	Standardized Representative Questions
1. Cognitive salience	“Even if you are not using the Internet/game/social network service (SNS), do you spend a lot of time thinking about the Internet/game/SNS or planning what to do next?”
2. Withdrawal	“Do you experience restlessness, irritability, depression, anxiety, sadness, etc. when you reduce, stop, or do not allow Internet/game/SNS?”
3. Tolerance	“Do you want to spend more Internet/game/SNS time, find more interesting things, or use better equipment such as cell phones or computers to make it feel as fun as before?”
4. Difficulty in regulating use	“Do you feel you should reduce Internet/game/SNS use, but cannot reduce the time you spend doing Internet/game/SNS?”
5. Decrease in other activities	“Because of the Internet/game/SNS, are you less interested in participating in other leisure activities such as hobbies or meeting friends?”
6. Persistent use despite negative consequences	“Despite negative consequences, such as lack of sleep time, being late to school or work, spending too much money, debating with other people, or neglecting important things, do you continue the Internet/game/SNS use?”
7. Deceiving	“Do you lie or hide how much time you spend on the Internet/game/SNS to your family or friends?”
8. Escape of negative feelings	“Do you use the Internet/game/SNS to avoid/relieve negative feelings?” “Do you use the game to forget unpleasant moods (e.g., helplessness, depression, guilt, anxiety, etc.)?”
9. Interference with role performance	“Have you ever been troubled or fallen out due to the use of Internet/game/SNS in your important interpersonal, career, and academic settings?”
10. Craving	“Do you have a strong desire to engage in activities such as using the Internet/game/SNS?” “If you want to play on Internet/game/SNS, is this desire difficult to tolerate?”

**Table 2:** Number of cases based on information from the Diagnostic and Statistical Manual of Mental Disorders-Fifth Edition (DSM-5) and International Classification of Diseases (ICD)-11 Internet game disorder (IGD) diagnosis criteria.

*n* (%)		ICD-11 Game Disorder (GD) Diagnosis Criteria		All
		Normal	GD	
**DSM-5 IGD Diagnosis Criteria**	**Normal**	115 (61.2)	0	115 (61.2)
	**IGD**	61 (32.4)	12 (6.4)	73 (38.8)
	**All**	176 (93.6)	12 (6.4)	188 (100)

**Table 3:** Internet usage characteristics of the three groups based on IGD diagnosis criteria.

*n* (%)		Normal (*n* = 115)		DSM5 (*n* = 61)		DSM5 + ICD11 (*n* = 12)		All (*n* = 188)		*x* ^2^
Internet content (percentage in all group)										4.19
1. Game		26 (13.8)		15 (8.0)		2 (1.1)		43 (22.9)		
2. SNS		9 (4.8)		2 (1.1)		0		11 (5.9)		
3. Other (YouTube, Africa TV)		9 (4.8)		5 (2.7)		0		14 (7.4)		
4. Combined	1.	71 (37.8)	51/11	39 (20.7)	23/7	10 (5.3)	8/2	120 (63.8)	82/20	
	2.		8/14		6/2		0/1		14/17	
	3.		12/46		10/30		2/7		24/83	
Internet device (percentage in all group)										8.359
PC		10 (5.3)		10 (5.3)		2 (1.1)		22 (11.7)		
Smartphone		55 (29.3)		18 (9.6)		4 (2.1)		77 (41.0)		
Other (tablet PC)		2 (1.0)		0		0		2 (1.0)		
Combination		48 (25.5)		33 (17.6)		6 (3.2)		87 (46.3)		

The left side is the primary Internet content and the right side is the secondary Internet content in the Internet content-combination.

**Table 4:** Psychiatric comorbidities of the three groups based on IGD diagnosis criteria.

*n* (%)	Normal (*n* = 115)	DSM5 (*n* = 61)	DSM5 + ICD11 (*n* = 12)	All (*n* = 188)	*x* ^2^
Psychiatric disorder (percentage within each group)					
Depressive dis.	12 (10.4)	12 (19.7)	4 (33.3)	28 (15.0)	6.229 *
ADHD	37 (32.2)	25 (41.0)	7 (58.3)	69 (36.7)	4.056
Anxiety dis.	3 (2.6)	1 (1.6)	0	4 (2.1)	0.447
OCD	2 (1.1)	1 (1.6)	0	3 (1.6)	0.20
ODD + CD	2 (1.7)	1 (1.6)	4 (33.3)	7 (3.7)	31.160 ***
Tic dis.	4 (3.5)	0	0	4 (2.1)	2.559
PTSD	1 (0.9)	1 (1.6)	0	2 (1.1)	0.375

* *p* < 0.05, *** *p* < 0.001. dis. = disorder, ADHD = attention deficit hyperactivity disorder, OCD = obsessive-compulsive disorder, ODD = oppositional defiant disorder, CD = conduct disorder, PTSD = post-traumatic stress disorder.

**Table 5:** Internet using behaviors of the three groups based on IGD diagnosis criteria.

*n* = 188	a. Normal (*n* = 115) M(SD) [95%CI]	b. DSM5 (*n* = 61) M(SD) [95%CI]	c. DSM5 + ICD11 (*n* = 12) M(SD) [95%CI]	*F*
Age when first used the Internet	7.47 (1.864) [7.13–7.82]	7.57 (2.384) [6.96–8.18]	7.42 (2.539) [5.80–9.03]	0.056
Age when first started internet games	8.06 (2.429) [7.61–8.51]	8.28 (2.138) [7.73–8.83]	8.42 (2.109) [7.08–9.76]	0.256
Age when first used smartphones	8.62 (2.130) [8.23–9.02]	8.87 (1.928) [8.38–9.36]	7.67 (1.875) [6.48–8.86]	1.734
Amount of time spent during weekdays (min/day)	248.78 (137.962) [223.30–274.27]	254.92 (146.579) [217.38–292.46]	290.00 (257.858) [126.16–453.84]	0.412
Amount of time spent during weekend (min/day)	361.57 (172.787) [329.65–393.48]	400.82 (186.148) [353.15–448.49]	477.50 (273.899) [303.47–651.53]	2.626

Age is ordinary age (Korean age).
